# Experiences with Policing among People Who Inject Drugs in Bangkok, Thailand: A Qualitative Study

**DOI:** 10.1371/journal.pmed.1001570

**Published:** 2013-12-10

**Authors:** Kanna Hayashi, Will Small, Joanne Csete, Sattara Hattirat, Thomas Kerr

**Affiliations:** 1 Urban Health Research Initiative, British Columbia Centre for Excellence in HIV/AIDS, St. Paul's Hospital, Vancouver, British Columbia, Canada; 2 Interdisciplinary Studies Graduate Program, University of British Columbia, Vancouver, British Columbia, Canada; 3 Faculty of Health Sciences, Simon Fraser University, Burnaby, British Columbia, Canada; 4 Global Drug Policy Program, Open Society Foundations, London, United Kingdom; 5 Thai AIDS Treatment Action Group, Chatuchak, Bangkok, Thailand; 6 Faculty of Medicine, University of British Columbia, Vancouver, British Columbia, Canada; University of Colorado, United States of America

## Abstract

**Background:**

Despite Thailand's commitment to treating people who use drugs as “patients” not “criminals,” Thai authorities continue to emphasize criminal law enforcement for drug control. In 2003, Thailand's drug war received international criticism due to extensive human rights violations. However, few studies have since investigated the impact of policing on drug-using populations. Therefore, we sought to examine experiences with policing among people who inject drugs (PWID) in Bangkok, Thailand, between 2008 and 2012.

**Methods and Findings:**

Between July 2011 and June 2012, semi-structured, in-depth interviews were conducted with 42 community-recruited PWID participating in the Mitsampan Community Research Project in Bangkok. Interviews explored PWID's encounters with police during the past three years. Audio-recorded interviews were transcribed verbatim, and a thematic analysis was conducted to document the character of PWID's experiences with police. Respondents indicated that policing activities had noticeably intensified since rapid urine toxicology screening became available to police. Respondents reported various forms of police misconduct, including false accusations, coercion of confessions, excessive use of force, and extortion of money. However, respondents were reluctant to report misconduct to the authorities in the face of social and structural barriers to seeking justice. Respondents' strategies to avoid police impeded access to health care and facilitated transitions towards the misuse of prescribed pharmaceuticals. The study's limitations relate to the transferability of the findings, including the potential biases associated with the small convenience sample.

**Conclusions:**

This study suggests that policing in Bangkok has involved injustices, human rights abuses, and corruption, and policing practices in this setting appeared to have increased PWID's vulnerability to poor health through various pathways. Novel to this study are findings pertaining to the use of urine drug testing by police, which highlight the potential for widespread abuse of this emerging technology. These findings raise concern about ongoing policing practices in this setting.

*Please see later in the article for the Editors' Summary*

## Abstract 2

Using thematic analysis, Kerr and colleagues document the experiences of policing among people who inject drugs in Bangkok and examine how interactions with police can affect drug-using behaviors and health care access.

*Please see later in the article for the Editors' Summary*

## Editors' Summary

**Background:**

In many countries, the dominant strategy used to control illegal drugs such as heroin and methamphetamine is criminal law enforcement, a strategy that sometimes results in human rights abuses such as ill-treatment by police, extrajudicial killings, and arbitrary detention. Moreover, growing evidence suggests that aggressive policing of illicit drug use can have adverse public-health consequences. For example, the fear engendered by intensive policing may cause people who inject drugs (PWID) to avoid services such as needle exchanges, thereby contributing to the HIV/AIDS epidemic. One country with major epidemics of illicit drug use and of HIV/AIDS among PWID is Thailand. Although Thailand reclassified drug users as “patients” instead of “criminals” in 2002, possession and consumption of illicit drugs remain criminal offenses. The 2002 legislation also created a system of compulsory drug detention centers, most of which lack evidence-based addiction treatment services. In 2003, the Thai government launched a campaign to suppress drug trafficking and to enrol 300,000 people who use drugs into treatment. This campaign received international criticism because it involved extensive human rights violations, including more than 2,800 extrajudicial killings of suspected drug users and dealers.

**Why Was This Study Done?:**

Drug-related arrests and compulsory detention of drug users are increasing in Thailand but what is the impact of current policing practices on drug users and on public health? In this qualitative study (a study that aims for an in-depth understanding of human behavior), the researchers use thematic analysis informed by the Rhodes' Risk Environment Framework to document the social and structural factors that led to encounters with the police among PWID in Bangkok between 2008 and 2012, the policing tactics employed during these encounters, and the associated health consequences of these encounters. The Risk Environment Framework posits that a range of social, political, economic, and physical environmental factors interact with each other and shape the production of drug-related harm.

**What Did the Researchers Do and Find?:**

Between July 2011 and June 2012, the researchers conducted in-depth interviews with a convenience sample (a non-random sample from a nearby population) of 42 participants in the Mitsampan Community Research Project, an investigation of drug-using behavior, health care access, and drug-related harms among PWID in Bangkok. Respondents reported that policing activities had intensified since rapid urine toxicology screening became widely available and since the initiation of a crackdown on drug users in 2011. They described various forms of violence and misconduct that they had experienced during confrontations with police, including false accusations, degrading stop and search procedures, and excessive use of force. Urine drug testing was identified as a key tool used by the police, with some respondents describing how police caused unnecessary humiliation by requesting urine samples in public places. It was also reported that the police used positive test results as a means of extortion. Finally, some respondents reported feeling powerless in relation to the police and cited fear of retaliation as an important barrier to obtaining redress for police corruption. Others reported that they had adopted strategies to avoid the police such as staying indoors, a strategy likely to impede access to health care, or changing their drug-using behavior by, for example, injecting midazolam rather than methamphetamine, a practice associated with an increased risk of injection-related complications.

**What Do These Findings Mean?:**

These findings suggest that the policing of PWID in Bangkok between 2008 and 2012 involved injustices, human rights abuses, and corruption and highlight the potential for widespread misuse of urine drug testing. Moreover, they suggest that policing practices in this setting may have increased the vulnerability of PWID to poor health by impeding their access to health care and by increasing the occurrence of risky drug-using behaviors. Because this study involved a small convenience sample of PWID, these findings may not be generalizable to other areas of Bangkok or Thailand and do not indicate whether police misconduct and corruption is highly prevalent across the all police departments in Bangkok. Nevertheless, these findings suggest that multilevel structural changes and interventions are needed to mitigate the harm associated with policing of illicit drug use in Bangkok. These changes will need to ensure full accountability for police misconduct and access to legal services for victims of this misconduct. They will also need to include ethical guidelines for urine drug testing and the reform of policies that promote repressive policing and compulsory detention.

**Additional Information:**

Please access these websites via the online version of this summary at http://dx.doi.org/10.1371/journal.pmed.1001570.

- This study is further discussed in a *PLOS Medicine* Perspective by Burris and Koester
- Human Rights Watch, a global organization dedicated to defending and protecting human rights, has information about drug policy and human rights, which includes information on Thailand
- The Global Commission on Drug Policy published a report in June 2012 entitled “The War on Drugs and HIV/AIDS: How the Criminalization of Drug Use Fuels the Global Pandemic” (available in several languages)
- The Global Commission on HIV and the Law published a report in July 2012 entitled “HIV and the Law: Risk, Rights and Health” (available in several languages), the Open Society Foundations have prepared a briefing on this report
- More information about the Mitsampan Community Research Project is available

## Introduction

In many countries, repressive criminal law enforcement is the dominant strategy for drug control [1]. However, a growing body of research has suggested that aggressive policing such as crackdowns has not reduced drug use and has instead contributed to adverse public health consequences, including epidemics of HIV infection among people who inject drugs (PWID) [2],[3]. For example, the fear generated by intensive policing may cause PWID to retreat into remote or hidden settings and avoid services such as needle exchange that can help protect them from HIV infection [4]–[6].

Thailand has been contending with longstanding dual epidemics of illicit drug use [7] and HIV/AIDS among PWID [8]. The Thai government has regarded the widespread use of illicit drugs as a “national crisis” and called upon all sectors of society to unite as a “national force” to combat this crisis [9]. The repressive drug prohibition approach has been closely linked with the HIV epidemic. For example, a large number of studies have identified incarceration as an independent risk factor of HIV infection among PWID in this setting [10]–[13]. Although the 2002 *Narcotic Addict Rehabilitation Act B.E. 2545* reclassified people who use drugs as “patients” instead of “criminals,” possession and consumption of illicit drugs remain criminal offenses [14]. Further, the new legislation created a system of compulsory drug detention centres (referred to as *bangkap bambat* or “forced treatment”) where those charged with illicit drug use are confined to undergo “rehabilitation.” However, the majority of these centres are run by the military and lack evidence-based addiction treatment services [15]. Recently, these centres have attracted strong criticism, as 12 agencies of the United Nations (UN) urged governments around the world to close down such centres [16].

Since the launch of this system, the Thai government has implemented a series of police crackdowns focused on illicit drug use and expanded the system [15]. Most notably, in 2003, a “war on drugs” campaign was launched to supress drug trafficking and to enrol 300,000 people who use drugs into treatment, mostly through compulsory drug detention. Being pressured to meet mandatory arrest quotas and encouraged to use “harsh” means during arrests, the police reportedly engaged in abusive practices, including more than 2,800 extrajudicial killings of suspected drug dealers and users [17],[18]. Between 2008 and 2011, the number of people who use drugs that were targeted to undergo rehabilitation programs increased from 60,000 in 2008 to 400,000 in 2011 [9],[19]–[21]. Although the policy emphasizes voluntary access to drug treatment, compulsory drug detention centres remain the principal means to enrol people who use drugs in treatment. In 2010, over 60% of those in drug treatment were placed in such centres [22]. Between September 2011 and August 2012, the number of drug-related arrests increased by 14% compared to the previous year, and more than 330,000 persons were arrested [23].

Despite concern that recent drug policy developments may have marked a return to the old drug war [24], few studies have investigated current policing practices in Thailand. Although a number of public health evaluations of aggressive policing have been undertaken in other countries [5],[6],[25]–[27], it is unknown to what extent these findings are applicable to Thailand, given that the legal, structural, and social environment surrounding drug use differs across settings. Therefore, this qualitative study sought to examine PWID's recent experiences with policing in Bangkok, Thailand. Our specific study objectives were to characterize the social and structural factors leading to encounters with the police among PWID, and to analyse policing tactics employed during these encounters, particularly with respect to international human rights norms for policing, as well as to identify the associated health consequences.

## Methods

### Ethics Statement

This study was approved by the research ethics boards at Chulalongkorn University and the University of British Columbia.

### Data Collection

A qualitative descriptive approach [28] was the methodological orientation underlying the study. The study was also informed by Rhodes' Risk Environment Framework [29],[30], which encourages consideration of social, structural, and environmental drivers of drug-related harm, and international human rights norms for policing. Qualitative data for this study were generated through in-depth interviews with PWID participating in the Mitsampan Community Research Project (MSCRP), a collaborative research effort involving the Mitsampan Harm Reduction Center (a peer-run drop-in centre in Bangkok, Thailand), Thai AIDS Treatment Action Group (Bangkok, Thailand), Chulalongkorn University (Bangkok, Thailand), and the British Columbia Centre for Excellence in HIV/AIDS/University of British Columbia (Vancouver, Canada). Launched in 2008 [31], this serial cross-sectional mixed-methods study aims to investigate drug-using behaviour, health care access, and drug-related harms among PWID in Bangkok. The present study was conducted as part of the larger qualitative study that sought to explore PWID's experiences with policing, compulsory drug detention centres, and access to HIV testing and care.

Between July 2011 and June 2012, semi-structured in-depth interviews were conducted with 48 PWID in Bangkok. Potential respondents were recruited face-to-face from the concurrent quantitative arm of the project [32] as well as through peer-based outreach efforts and word-of-mouth, and were invited to attend the Mitsampan Harm Reduction Center or O-Zone House (another drop-in centre in Bangkok) in order to participate in the study. Adults residing in Bangkok or in adjacent provinces who had injected drug(s) in the past 6 months were eligible for participation. Sampling methods were purposive, and efforts were made to attain balance in age, gender, and HIV serostatus and to recruit individuals who had encounters with police in the past 3 years and/or those who had been in compulsory drug detention centres in the past 5 years.

Two bilingual Thai research assistants (including the study's fourth author, SH) were trained by KH (a PhD student) and TK (a public health researcher) to conduct interviews in Thai based on a semi-structured interview guide (Text S1). Both interviewers were women, had master's degree in health-related disciplines, and have been involved in the MSCRP as local research assistants prior to the present study. The pre-existing relationship with the study population facilitated rapport between respondents and interviewers. Both interviewers had long been committed to work that promotes evidence-based HIV-focused policies and interventions, as well as the human rights of affected populations. With regard to encounters with police, the interview guide sought to elicit discussions about: under what circumstances police approached respondents; police's search-and-arrest procedures during the most recent encounters with police; any negative and positive experiences with police; how the police identify and detain PWID in general; respondents' reactions to any police misconduct and abuse; the impacts of policing on respondents' drug use patterns, health care access, and daily lives. The interview guide was reviewed by local community research partners, which served to fine-tune the questions. Interviewers were also encouraged to employ additional questions and probes to explore each individual respondent's experience.

Throughout the data collection process, the research team discussed the content of interview data as well as the focus and direction of subsequent interviews. Data collection was continued until data reached a point of saturation (new respondents' narratives reiterated points made previously). All respondents provided informed consent and were interviewed by the two interviewers. No respondents dropped out from interviews. All interviews were conducted in private rooms at the Mitsampan Harm Reduction Center and O-Zone House. During the interviews, only the respondent and interviewer were present in the room. Interviews lasted between 40 and 90 minutes and were audio-recorded. Upon completion of the interview, respondents received a stipend of 450 Thai Baht (approximately US$15), and interviewers took brief notes of the interviews summarizing the key topics covered and any problems identified during the interviews. There were no repeat interviews. While transcripts were not returned to respondents for comments, interviewers summarized the main points during the interview, which provided opportunities for respondents to confirm whether interviewers properly captured the content of the respondents' narratives.

### Data Analysis

All audio-recorded interviews were transcribed verbatim in Thai and translated into English. The interviewers who were bilingual in Thai and English and who have developed familiarity with terms used among local PWID reviewed the translated transcripts for accuracy. Further, a native English-speaking proof-reader with an excellent knowledge of both Thai and English also verified the English transcripts for grammatical accuracy and nuance by comparing the English transcripts with Thai transcripts and audio-files.

In-depth interview data were analysed to identify the situational factors leading to police encounters as well as to identify policing tactics employed during these interactions, particularly with respect to international human rights norms for policing. We also analysed respondents' actions and behaviours after the police encounters, and any subsequent health consequences. All data were entered into Atlas.ti (version 6.2), software designed to assist qualitative data management and analyses. Data analysis was informed by the Risk Environment Framework [29],[30], which posits that a range of social, political, economic, and physical environmental factors interact each other and shape the production of drug-related harm. Given that the past “war on drugs” campaign resulted in numerous human rights abuses in this setting, the analysis was also informed by the work of Jürgens et al. [33], which asserts that rights violations also constitute core features of risk environments surrounding drug use.

Data analysis was conducted inductively, employing a multi-step thematic analysis. On the first pass, KH created an initial set of codes. Subsequent reviews involved refining the codes and assigning data segments to categories with substantive input from other co-authors, including WS, JC, and TK who had extensive experience in qualitative investigations of the impacts of policing among people who use drugs [5],[34]. The analysis considered the range and diversity of respondents' experiences, as well as negative evidence in each category of experience. Finally, the data were grouped into three parts in chronological order: circumstances of police confrontations, police violence and misconduct, and PWID's reactions to drug policing practices. We did not seek feedback from respondents on the study findings for several reasons, including practical challenges with following up all respondents (e.g., the study protocol did not allow us to record respondents' identifying information), the potential representational problems associated with this technique (e.g., some respondents' experiences may not be relevant to others) [35], and the consistency between the findings and our past quantitative work [36]–[40] and observational experiences with local PWID over the past 5 years.

## Results

In total, 42 PWID were interviewed for this study, including 17 (40.5%) women. The median age was 35.5 years (range: 23–52 years). Table 1 summarizes the respondents' demographic characteristics, drug-using behaviour, and self-reported HIV status. All respondents reported interactions with police during the three years before the interviews. As shown below, we used verbal counting to highlight regularities, peculiarities, and idiosyncrasies in the data. In doing so, we operationally defined “many” and “common” as something reported by half or more of the respondents and “some” and “a few” as something reported by less than one-third of the respondents. However, inferences of generalizability from these terms are discouraged.

**Table 1:** Sample characteristics (*n* = 42).

Characteristic	*n* (%)
Female gender	17 (40.5%)
Age:	
≤30 years	7 (16.7%)
31–40 years	21 (50.0%)
>41 years	14 (33.3%)
Drugs most frequently injected^a^:	
Midazolam	30 (71.4%)
Heroin	17 (40.5%)
Methamphetamine	13 (31.0%)
Crystal methamphetamine	10 (23.8%)
Methadone	4 (9.5%)
Self-reported HIV seropositivity	14 (33.3%)

^a^ Refers to the 6 months prior to the interview. Multiple answers were allowed.

### Circumstances of Police Confrontations

Various factors surrounding and leading to police confrontations were identified from respondents' accounts and grouped into four main themes including drug policies and laws, financial incentives within policing structures, police surveillance methods, and individual characteristics. Respondents' narratives indicated that many of these main themes as well as sub-themes interacted with each other and could result in various forms of police misconduct and violence as described in the following section. Under the theme of drug polices and laws, several sub-themes were identified, including the 2011 large-scale police crackdown; changes in drug laws that enabled police officers to use rapid urine drug testing; mobilization of civil volunteers in drug policing; and the focus on young people. Sub-themes of the police surveillance methods were further categorized into those specific to physical environments (i.e., roadside checkpoints, vicinities of methadone clinics, and police raids on one's home) and social environments (i.e., undercover police operations, police informants, and civil volunteers). Sub-themes within the individual characteristics included age, prior contact with the local police, and having visible signs of drug use (e.g., needle marks and tattoos).

Respondents noted that policing during the past 3 years was experienced as recurrent waves of crackdowns on people who use drugs. It was reported that policing activities had noticeably intensified since rapid urine toxicology screening became widely available to police.

*R:* [The police] *have become more repressive these days. Now they insist on urine testing! In the past, all they did was to check our arms to see if we had needle marks. Okay, that's like red-handed. But now they can arrest us for having drugs in our body. Is my urine illegal now? Isn't this too much?* (Respondent #27, female, age 32)

Respondents also indicated greater police pressure since the government initiated a large-scale crackdown on people who use drugs in 2011 [41]. In particular, the local police appeared to be pressured to make arrests towards the end of the year 2011 when they needed to submit the arrest records to the authority.

*R: Towards the end of the year [2011], the police needed to submit records to higher-ups, right? So, they wanted to look impressive. This police officer knew us for long time. He came to us and said, “Hey! You come help with the nation!” Then, he put us in a jail for nothing for a week. He said it was for the nation.* (Respondent #35, female, age 50)

During the 2003 “war on drugs” campaign, the police had to fill arrest quotas and were rewarded for making drug-related arrests [18]. Although it is unknown from publicly available information whether mandatory arrest quotas were still in place under the subsequent drug policies, according to the Office of the Narcotics Control Board of Thailand (A. Sirisabphaya, personal communication, April 9, 2013), a cash reward system does remain in place for confiscation of drugs. Some respondents suggested that financial incentives stimulated the police to make drug-related arrests.

*R: There is a price on each person's head. It's like a quota coming down to each police station. And they get money when they make an arrest … It's their system. I really don't think it's right. It's just for the money.* (Respondent #16, male, age 35)

Respondents described various overt and covert surveillance methods employed by police officers to identify people who use drugs. Many police officers reportedly wore plain clothes at work. A couple of respondents also reported that officials of the Office of the Narcotics Control Board of Thailand engaged in arresting drug dealers. Although many respondents claimed they could immediately identify a plain-clothes officer by his/her appearance, some respondents reported confusion because these plain-clothes officers sometimes demanded a search without identifying themselves as police, and at the same time would not show identification when asked. One account noted that in this situation an unscrupulous person could pretend to be a policeman and position himself to rob a person who uses drugs.

*R*: *When I was at a methadone clinic, three young guys came to me and said, “Won't you sit down and talk to the police?” They were actually new police officers that I'd never seen before. So, I asked, “Sit down for what? Can I see your ID?” Then, they said, “Hah! Are you a smart ass?” I'd met someone who pretended to be a police officer and robbed me of some stuff. That's why I asked for ID. But he said I was a smart ass!* (Respondent #24, male, age 46)

Many respondents cited roadside checkpoints as a police surveillance method. These checkpoints were set up in diverse locations at various times but particularly at night and in “red zones,” which denote districts in which many drug dealers and people who use drugs are believed to congregate, including “slum-like neighbourhoods” (respondent #1, male, age 37).

*R: These days the police have increased their efforts to find and arrest us. My place is in a red zone. It's a dangerous zone. They put up campaign signs and often set up checkpoints.* (Respondent #15, male, age 43)

Respondents also reported that the police were present around methadone clinics, presumably to take advantage of the volume of people who use drugs coming and going from these venues. A patient in methadone treatment reported that the police threatened to send him and other patients to compulsory drug detention centres:

*R: After taking methadone, I was sitting in front of the clinic with my friends. Then, 3–4 police officers came, saying “Hey, you! Come over here! If we find drugs in your urine, what do you want us to do?” I said, “Sir, you can't find anything because we don't use yaba or ice* [i.e., methamphetamines]. *We're patients taking methadone everyday.” Then, they said, “If your urine comes out positive, you'll be sent to ‘treatment’* [i.e., compulsory drug detention centres] *right away!” So, I said, “Such ‘treatment’ can't treat drug users.” Then, they said, “Bastard! You think you're a smart ass?”* (Respondent #25, male, age 41)

Respondents perceived that several individual factors made them “look like drug users” or otherwise increased the chance of being subjected to stop-and-search procedures by the police. These factors included being known to the local police as a person who uses drugs (e.g., those with criminal records), being young, having visible tattoos or needle marks, and looking nervous. Some respondents shared a belief that tattoos signified that a person had been in prison and were indicative of being a drug offender.

*I: Why do you think the police stopped you three times in a month?*

*R: I have lots of tattoos. The police like to keep an eye on guys with tattoos. …People think we've been in prison. Good people don't have tattoos. Only ex-cons do!* (Respondent #13, male, age 36)

In addition to roadside stop-and-search surveillance, the police also reportedly relied on information supplied by people who use drugs or local residents to identify potential offenders. For example, some respondents were forced by police to identify known drug dealers (“to be a finger for the police”). However, all of them reported that they refused to “be a finger” out of fear of retaliation and distrust from drug dealers and other people who use drugs.

*R*: [The police officer] *also wanted me to be a ‘finger’ for him. If I did that, he would let me go. It was like an exchange. So, I said that I'd give him information on where the drug dealer lived. But I didn't give him real information. I just randomly picked a house in the neighbourhood.* (Respondent #35, female, age 50)

As the Thai drug authorities have ordered local authorities to engage civilians in identifying people who use drugs [9],[22],[42], respondents' narratives indicated that anyone in the neighbourhood could be “a finger for the police.” Also, it was reported that during crackdowns, local residents volunteered to assist police officers with finding drug offenders.

*R: I was standing on a street, waiting for the stuff* [drugs]. *Then, a big bus drove by. There were about 10 police volunteers in there. They said, “Stay still! Don't move!” Then, they took me to a police station to do a urine test.*

*I: But they weren't police officers, were they? Why did you feel that you had to follow them?*

*R: They were locals and called themselves volunteers. And it was a crackdown. I already knew how it would go.* (Respondent #30, female, age 33)

One consequence of being identified by the local police as a person who uses drugs was a police raid on one's home, sometimes after midnight. Some respondents who were raided in their homes tended to experience it more than once.

*R: …They know where I live! They know they will get me. So, they keep coming… I'm scared. I was sent to prison eight times! I don't wanna go there again. I've never been arrested outside my house. They always get me at my house.*

*I: Why do the police come to your house so often?*

*R: It could be because some people reported on me. I don't know. But when they come, they say, “People reported on you. So, we are here to arrest you!” …Some people in my community love me, but others hate me.* (Respondent #17, male, age 23)

*R: …5–6 undercover police officers. They came at night. I was going to bed at that time. They break in whenever they want! They even climbed over the wall!*

*I: They weren't wearing a uniform, right? Weren't you surprised at having 5–6 strangers climbing into your house? Didn't you think that they were thieves?*

*R: Thieves wouldn't climb into my house. But the police would do. So, I knew they had to be the police.*

*I: When they came in, what did they say?*

*R: They just told me to put on my clothes and come with them to the police station to do a urine test.* (Respondent #18, male, age 29)

### Police Violence and Misconduct

Respondents described various forms of police violence and misconduct that they experienced first-hand. Main themes in this section included: false accusations, degrading stop-and-search procedures, urine drug testing, extortion of money, coerced confessions, and excessive use of force. In particular, urine drug testing was identified as a key tool used by the police, and the provision of test results appeared to be closely associated with other forms of police violence and misconduct (e.g., extortion of money and excessive use of force). Urine drug testing also served as a sub-theme of degrading stop-and-search procedures.

Some respondents known to local police officers as people who use drugs reported that they were coerced into “helping” the country by admitting guilt to false charges.

*R: They said, “Help the nation with some work! What charge do you want?”*

*I: Did they find any drugs on you when they said that?*

*R: No! But I knew what charge I should go for. So, I picked the one with the minimum sentence.*

*I: Did they let you pick a light charge?*

*R: Mostly they do, because that way, they can arrest you again after you've been released.* (Respondent #26, male, age 36)

Many respondents reported that police would immediately search their bodies or belongings, often in degrading ways. Possession of drug paraphernalia was experienced by some respondents as grounds for arrest, despite the National Police Office's memorandum instructing that it should not be done [43]. In the absence of illicit drugs or drug paraphernalia, many respondents were forced to undergo urine toxicology testing. Stop-and-search and drug testing procedures typically took place in public places, which some respondents felt caused unnecessary humiliation:

*R: There were people walking around. They wanted me to pee in a corner. There was nowhere to hide. Isn't that nasty? It's not at all proper. They could have let me find a more discreet place. Passers-by looked at me and knew what I was doing. Women giggled.* (Respondent #10, male, age 35)

*R: First,* [two male police officers] *searched inside my bag. There was nothing in it. Then, they told me to take off my bra, right in front of the Soi* [i.e., street]! (Respondent #29, female, age 23)

Respondents reported that police attempted to extort money from them, particularly when the results of drug testing were positive. In many cases, the respondents were presented with an opportunity to provide a bribe and negotiate with police for lighter charges or avoid the charge altogether. The negotiation was initiated by either the person detained or the police, who may cite a specific monetary value or goods (e.g., a bottle of whiskey) in return for a bribe.

*R: They asked, “How much money do you have?” I asked, “Will a thousand do?” They said, “Two thousand.” So I gave them 2,000 baht* [i.e., approximately US$ 66]. *Then they told me to fill out a form saying that I was arrested for not carrying my driver's licence. Because they brought me to the police station, they had to charge me with something.* (Respondent #21, male, age 30)

*R: If your urine turns purple, but you have money and want to negotiate with them, the urine is magically no longer purple. This is what happens in most cases.* (Respondent #1, male, age 37)

Many respondents also stated that they were falsely accused, had evidence planted on them by police, or were coerced into confessing to a crime that they did not commit (e.g., theft). Some respondents reported that they were compelled to sign a blank sheet and were not informed of the charges they faced until at a later stage.

*R: He just handed me a letter to sign. I thought it was paperwork for a urine test. Then he said, “Here! The charge has been changed from drug use to stealing. Otherwise you would have been sent away for a long time this time. You wouldn't have the money to bail yourself out. Just think of it as a favour. Or do you want me to charge you with something heavier?”* (Respondent #27, female, age 32)

Excessive use of force was reported as another means employed by police to extract a confession from a detainee. Many of the respondents who experienced this form of violence asserted that these confessions were often false. The methods used by police included beating or kicking suspects, sometimes combined with a physical restraint (e.g., handcuffs), electric shock, and being soaked in ice water.

*R: When we arrived at the precinct, they gave us a blank sheet of paper to sign. We had the ‘blank paper treatment’ before. So we knew! I told them, “Sir, we can't sign on a blank sheet of paper. If you don't let us read it first, we won't sign.” Then, six or seven officers took me into a small room. It was a sound-proof glass room…Then, they kicked me. Thud! Thud! Then, they wrapped me with a blanket and blasted the air conditioner. They soaked me with icy water… I was shaken up so badly. They did that to me for three hours.* (Respondent #25, male, age 41)

### PWID's Reactions to Policing

The data regarding PWID's reactions to policing were grouped into two categories: barriers to seeking justice and strategies to avoid the police. In the first category, two main themes were identified: social factors and factors related to the judicial system and processes. Sub-themes under the social factors theme included police corruption and feeling powerless in relation to police, whereas sub-themes related to the judicial system and processes included poor availability of legal services, slow judicial proceedings, fear of detention, and being a drug offender. Regarding the strategies to avoid the police, main themes included restricting one's activities, moving out of “red zones,” changing drug-using behaviour, or not employing any strategies. Retreating into one's house and reduced access to health care were identified as sub-themes of restricting one's activities. Sub-themes related to drug-using behaviour included resorting to discreet locations for drug use, hurried injections, refraining from using methamphetamines, and changing types of drugs consumed. Sub-themes assigned to the last theme included anxiety, fatalism, and drug use as stress coping.

Despite having experienced police abuse, respondents showed reluctance to report these experiences to the authorities or seek justice. Some respondents reported feeling powerless in relation to police, and felt discouraged and intimidated by the police officers' disregard for their rights:

*R: The police don't give you any respect. If you talk about your rights, you'll just end up getting hurt. Even though the law supports your rights, the police will think you're a know-it-all. They may have it out for you.* (Respondent #34, male, age 35)

Other respondents reported police corruption and fear of retaliation from the police as being important barriers to obtaining redress.

*R: In my district, people from this political party abuse the power. If they don't like anyone, they would tell the police to take care of the person. And the police would do anything to put this person in a jail. They can do it even when this person hasn't committed any crime. They are much more powerful than we are. To make it simple, they have money. How can we fight against them? Do we have money? No.* (Respondent #35, female, age 50)

*R: I wanted to report it* [that her partner was beaten by police during the interrogation] *to the Chief Inspector. But my boyfriend and his mom told me to just let it go. They were afraid that it wouldn't end well if I reported it to the police.*

*I: What do you mean?*

*R: They were afraid that the police might think that we brought too much trouble. The police might do something to us.* (Respondent #36, female, age 37)

In addition, respondents reported barriers related to the judicial system and processes, including limited knowledge about or access to legal services, slow judicial proceedings, and fear of detention while awaiting a trial. One respondent recounted a 3-year-long court fight against a false accusation. Furthermore, some respondents believed that a previous drug conviction meant that they could not win a court case over police misconduct or prove their innocence. One respondent reported that a court-appointed lawyer even advised that he accept a false charge rather than fight it:

*R: The court-appointed lawyer said that a confession would make things easier, but if I chose to fight, it would be a long fight. He didn't have any other suggestions for me. He probably thought I wasn't in a position to fight this false charge because I had a previous record* [drug-related charges]. *So, I was doomed to lose.* (Respondent #9, male, age 34)

As a result of numerous and repeated negative interactions with the police, many respondents employed strategies to avoid the police. Common strategies included retreating to one's house or a new location outside “red zones” and making changes to drug use behaviours. Many of these tactics had negative impacts on respondents' health and well-being.

The simplest strategy for avoiding police was to refrain from going outside where one could be subjected to police scrutiny. This strategy often impeded respondents' access to health care, including methadone clinics.

*I: Are there any other reasons that make you feel like you don't want to go to the doctor?*

*R: Yes, I'm scared of the police checkpoints in the area. I could go during rush hour. But if it's a little later, I don't want to go.* (Respondent #21, male, age 30)

*R: When they* [the police] *were campaigning against drug use, we couldn't even get into the methadone clinic. We had to wait. And we couldn't hang around there and let them see us either. They would often wait for us along the routes we use. I've run into trouble with them two or three times.* (Respondent #5, male, age 50)

Some respondents also reported that they changed the venues where they used drugs, resorting to injecting drugs alone in more discreet locations, while others engaged in hurried injections out of fear of being detected by the police.

*R: I inject drugs mostly at gas stations. I hurry in and hurry out because it's dangerous. Sometimes the police check these places, and if the staff at the gas station sees me go in there* [to the bathroom] *for a long time, they may call the police. So, I have to do it fast.* (Respondent #15, male, age 43)

Respondents noted that police actions also sometimes led them to change the types of drugs they consumed. Several respondents believed that the rapid urine screening kits used by the police detected the presence of methamphetamines only, so they stopped using methamphetamines and shifted to other drugs, including midazolam, a short-acting benzodiazepine that can be obtained from private clinics.

*R: I've definitely stopped using ice* [crystal methamphetamine] *and meth. Urine tests only test for meth. So now I only inject heroin and Dormicum* [midazolam]. (Respondent #14, male, age 32)

Finally, some respondents did not actively employ any strategies to avoid police confrontations but felt concerned and anxious about the intensive and endless police pressure. These individuals reported either becoming fatalistic about the risk of police encounters and the associated harms or engaging in drug use to cope with the excessive stress.

*R: I'm paranoid about the police. Every day when I'm at home, I don't feel like going to bed… The “puyai”* [i.e., elders] *in my neighbourhood all know whether I have drugs or not, and the police will come and take me. …But I have to just let it go. Whatever happens happens.* (Respondent #18, male, age 29)

*R:* [The police] *stress me out. They make me feel like using* [drugs] *so that I can forget about them! It's as simple as that!* (Respondent #22, female, age 47)

## Discussion

The findings of this study suggest that policing of PWID in Bangkok has involved injustices, human rights abuses, and corruption. Consistent with a large body of literature from several settings [4],[6], findings also indicate that aggressive policing has compromised the health of PWID through various pathways.

This study has several limitations that should be noted when interpreting the findings. First, our findings are based on interviews with PWID who had direct encounters with police in the previous 3 years. Therefore, experiences and views of non-PWID or other PWID who did not confront police officers were not included. Second, because the respondents were asked to remember experiences they had up to 3 years ago, their narratives may have been affected by recall bias. Third, although efforts were made to attain a balance in demographic characteristics among the interviewees, we could not meaningfully reach sub-populations of PWID who also belonged to other vulnerable populations, including transgendered persons, migrants, and sex workers. Their experiences with police may be different from those reported here. The potential biases associated with our small convenience sample may limit the transferability of the findings. Lastly, it was not possible to determine from this analysis whether the police misconduct and corruption reported by respondents were highly prevalent and ongoing across the all police departments in Bangkok.

A notable finding of this study is evidence of harm related to the use of urine testing by police. In Thailand, the 2000 amendment of the *Narcotics Control Act* (Section 14) and the 2003 amendment of the *Narcotics Act* (Section 58/1) allowed law enforcement officers to perform drug testing on anyone upon the basis of “reasonable suspicion” [14]. According to the Office of the Narcotics Control Board of Thailand (A. Sirisabphaya, personal communication, March 28, 2013), the police use two kinds of rapid urine screening kits (one screening methamphetamines only and the other screening multiple drugs). Use of this tool empowered police to identify drug offenders even when they were not in possession of illicit drugs or in the act of using drugs. A recent survey of 435 community-recruited PWID in Bangkok reported that 27% of the sample had experienced urine drug testing by police during the past six months [38], suggesting widespread use of this technology by the police in this setting. The experiences recounted here indicate abusive use of this tool: some people were forced to urinate in public places, and the police reportedly used positive test results as a means of extortion. Furthermore, respondents believed that police tested only for methamphetamine, leading some to transition from methamphetamines to midazolam use. Midazolam injection is associated with elevated risk of severe injection-related complications, such as nerve and vascular injuries [32],[44]. Echoing earlier results from other police practices in Sydney, Australia [6], these findings suggest that potential gains from disrupting the use of certain illicit drugs may be offset by the harm associated with the misuse of other drugs, as policing forces changes in drug-using behaviours.

Our findings shed light on some social and structural factors contributing to the observed rights violations. Some respondents perceived increasing police pressure in 2011 when the Thai government substantially increased numerical targets for drug treatment enrolment [9]. The police are known to take the number of new admissions to drug treatment as an indicator of success of the drug policy [23], where drug “treatment” primarily means being sentenced to compulsory drug detention [22]. Respondents' accounts and available information indicated that financial incentives for policing have persisted. Given the low salaries of police officers in Thailand [45], these incentives may have promoted aggressive and corrupt policing practices. These features of Thai policing may help explain the frequent police raids on known PWID's homes, evidence planting, and false accusations. As suggested by some respondents, it may also be that the police intentionally charged people with lesser offenses so that they would be released and available for re-arrest to help inflate arrest figures. Evidence planting and false accusations represent a violation of the right to freedom from arbitrary arrest and detention under Article 9 of the International Covenant on Civil and Political Rights (ICCPR), which Thailand ratified in 1996. The results also indicated that the police used physical force to coerce confessions in some cases. According to a recent study, 37% of PWID in Bangkok interviewed reported having been beaten by police, most commonly during the interrogation process [37]. This practice is a violation of the rights to security of the person (Article 9) and to freedom from torture and cruel, inhuman, and degrading treatment (Article 7) under the ICCPR. These rights are also enshrined in the 2007 Constitution of the Kingdom of Thailand B.E. 2550 (Section 32) [46].

Consistent with studies from other countries [5], our findings indicated that police harassment near methadone clinics deterred some methadone patients from receiving treatment. This type of targeted police interference with essential health services is a violation of the right to the highest attainable standard of health enshrined in the International Covenant on Economic, Social and Cultural Rights (Article 12) [47], to which Thailand became a party in 1999.

In addition, the findings indicate that police corruption and other police misconduct have further contributed to PWID's vulnerability to drug-related harm. We identified extortion of money by the police as a main theme of the police violence and misconduct. This is consistent with a previous study from this setting reporting that half of PWID who reported having drugs planted on them paid police a bribe in order to avoid arrest [36]. Some respondents also reported arrests for syringe possession and needle marks; such arrests have been found in other settings to facilitate risky injection behaviour and impede access to health care [26],[48],[49]. Respondents' accounts that plain-clothes police did not always identify themselves as police were concerning, not only because this practice is unlawful under the *Narcotics Act* (Section 49) [14], but also because some respondents were reluctant to exercise their right to ask for identification. Furthermore, our findings highlight possible abuses associated with “deputizing” local residents to help the police identify alleged drug offenders. Particularly where police are not always in uniform, deputized civilians may be mistaken for police or may overstep their authority, leading PWID to flee, hide, or otherwise respond in ways that may be risky to them and those around them. Mobilization of civilians of this kind has also been reported in Laos and Vietnam, where many civilians (e.g., heads of villages) are compelled by local authorities to help achieve “drug-free” environments and submit people who use drugs to the police or compulsory drug detention centres [50],[51].

Our findings also suggest that policing practices may have disproportionately affected some persons, particularly former drug offenders. Even though there is no registration system for people who use drugs in Thailand, as there is in many eastern European countries [52], in practice the Thai police reportedly maintain “blacklists” of suspected or formerly convicted drug dealers and people who use drugs [18],[41],[43]. These lists seem to serve effectively as a registration system and similarly increase the vulnerability of those listed to police abuse and extortion [52]. In addition, respondents reported that police appeared to target people with tattoos, which are taken as markers of former incarceration. This practice likely violates the principle of non-discrimination under Article 26 of the ICCPR and Section 30 of the Thai constitution [46].

It is particularly concerning that many respondents who experienced police abuse were forced to bear this abuse in silence. In accordance with its international human rights obligations, Thailand has a legal framework that prohibits state corruption, prohibits the use in court of evidence obtained through unlawful means, and grants victims of torture the right to seek redress and compensation [53],[54]. However, investigations of police abuses committed under the 2003 drug war have not been completed [55], indicating a political unwillingness to bring the perpetrators of these heinous abuses to account. This may be contributing to respondents' reluctance to seek justice. Our findings underscore repeated calls for ensuring full accountability for police abuses [55]. Given the observed lack of knowledge and access to legal services, greater efforts should also be made to provide legal services to PWID in Bangkok. A recent review highlighted that legal services are often as important as health services in safeguarding the rights and well-being of people who use drugs [56].

Collectively, our findings suggest a need for multilevel structural changes and interventions to mitigate the harm associated with policing in Bangkok. These include abolishing numerical targets for drug treatment enrolment that effectively promote compulsory detention, banning financial incentives and blacklists to reduce the potential for abusive policing practices, establishing binding commitments from the police not to interfere with health and harm reduction services, and training police to understand harm reduction activities. Globally, the latter two interventions have been applied in many settings and have faced such challenges as high turnover among police, varied public perceptions of the role of police, and police corruption [4],[57]. In particular, reports from Vietnam documented that a macro-level drug policy that emphasized compulsory drug detention for drug control measures has undermined the effectiveness of these police interventions aimed at supporting harm reduction services [57].

Repressive policing of PWID is largely attributable to harsh application of criminal penalties to a wide range of drug offenses. In recent years, a number of countries have experimented with alternative regulatory frameworks for illicit drugs, including decriminalization of personal drug use [58]. Decriminalization has a number of potential benefits to public health, including reducing harms associated with incarceration and pretrial detention of people who use drugs. Given some emerging evidence of positive results [2],[59], these alternative frameworks are worth exploring in Thailand.

Lastly, clear ethical standards need to be established to safeguard against abusive practices related to the police's use of drug testing. While drug testing is increasingly used in many settings, including health care settings, workplaces, schools, and correctional facilities [60],[61], there is scant literature to inform humane and pragmatic guidelines for drug testing by police, which are sorely needed. In Europe, following the model of roadside breathalyzer alcohol tests, some countries have authorized roadside drug testing to identify drivers under the influence of drugs [62]. Many countries have legal provisions stipulating that sanctions should be based on the impairment of driving ability by substance abuse, not on the analytical detection of drugs in body fluid [63]. The majority of the countries recognize that any roadside testing procedure can be an intrusion into individual rights and take measures to respect voluntariness and privacy (e.g., using a sanitary van for urine tests) [62],[63]. These experiences, particularly valuing the voluntary nature of drug testing, may be useful to inform developing ethical and rights-friendly drug testing practices in Bangkok.

In sum, this study suggests that policing in Bangkok has involved various injustices, human rights abuses, and corruption, and policing practices in this setting appeared to have increased PWID's vulnerability to poor health through various pathways. Novel to this study are findings pertaining to the use of urine drug testing by police, which highlight the potential for widespread abuse of this emerging technology. These findings raise concern about ongoing policing practices in this setting and indicate an urgent need to ensure full accountability for police abuses and access to legal services among the victims of police abuses. Further, ethical guidelines for urine drug testing by police are needed, as are reforms of policies that promote repressive policing and compulsory drug detention. The Thai government should instead develop policies that encourage access to voluntary evidence-based drug treatments and incorporate evidence-based harm reduction approaches. Future research should explore the views and experiences of police officers in order to identify challenges and opportunities related to the implementation of these policy recommendations.

## Supporting Information

Text S1 **A semi-structured interview guide (excerpt).**
(PDF)

## Abbreviations

PWID: people who inject drugs
