# Supplementary material for: Experiences with Policing among People Who Inject Drugs in Bangkok, Thailand: A Qualitative Study
Source: PLoS Med. 2013 Dec 10;10(12):e1001570. doi: 10.1371/journal.pmed.1001570 (PMC3858231; doi:10.1371/journal.pmed.1001570)
Supplement: Text S1 — A semi-structured interview guide (excerpt). (PDF) [file pmed.1001570.s001.pdf]

Text S1

Mitsampan Community Research Project 2011
Interview Guide for Qualitative Interviews
(Excerpt)

Topic Area II: Encounters with Police

**Question:**	**Probes:**
Now I'd like to ask you about your encounters with police. Can you describe the last time you encountered with police?	When and where? What were the circumstances?<br>Why do you think they approached you?<br>Why did the police say that they stopped you?<br>How did the police treat you?<br>If police arrested you, on what charges were you arrested?
Have you ever had any negative experience when encountering with police? If so, can you tell me what happened?	When? In what circumstances?<br>**Probe for various types of police abuse:**<br>Verbal abuse?<br>Harassment?<br>Physical violence (e.g., beaten)?<br>Had drugs planted on you?<br>Paid bribes?<br>Police confiscated your drugs, syringes, or other injection equipment?<br>Arbitrarily arrested (i.e., police arrested you without telling you on what charge they arrested you or without any evidence of the charge)?<br>Coerced into false confession?
If you have had other negative encounters with the police, could you please describe those?	Where? When? What were the circumstances?
What did you do about it?	Asked for legal assistance? If not, why?
Have you ever had any positive experience when encountering with police? If so, can you tell me what happened?	When? In what circumstances?<br>Did they refer you to:<br>- harm reduction centers?<br>- drug treatment centers?
How did your encounters with police impact your life afterwards?	How did it influence:<br>- your place to stay?<br>- your relationship with your family?<br>- your interactions with the community you live in?

	- your access to health care services?<br>- your access to employment and jobs ?
How did your encounters with police affect your drug use patterns?	**Probe for:**<br>Types of drugs you use?<br>Sources of drugs you use?<br>Frequency of drug use?<br>The way you use drugs (e.g., shifted to injecting into groin or deep vein injecting)?<br>Rushing injection?<br>Places where you use drugs<br>The time when you use drugs?
How do your encounters with police and police presence shape your access to health care?	
How do the police identify and detain drug users?	How do the police know who may be a drug user?<br>Where do the police look for drug users?<br>**Probe for:**<br>Near health service outlets?<br>Near centers where methadone is distributed?<br>Near places where syringes can be obtained?<br>Near known drug market locations?
Can you tell me anything you are doing to avoid encountering with police, if any?	Are there places that you avoid so that you do not encounter police? If so, what are these places?
Would you like to say anything else about this topic?	
